# Supplementary figures and images for: Comparing regular expression and machine learning approaches to predict immigrant status from primary care electronic medical record data in Ontario, Canada
Source: PLOS Digit Health. 2026 Apr 17;5(4):e0001336. doi: 10.1371/journal.pdig.0001336 (PMC13089691; doi:10.1371/journal.pdig.0001336)

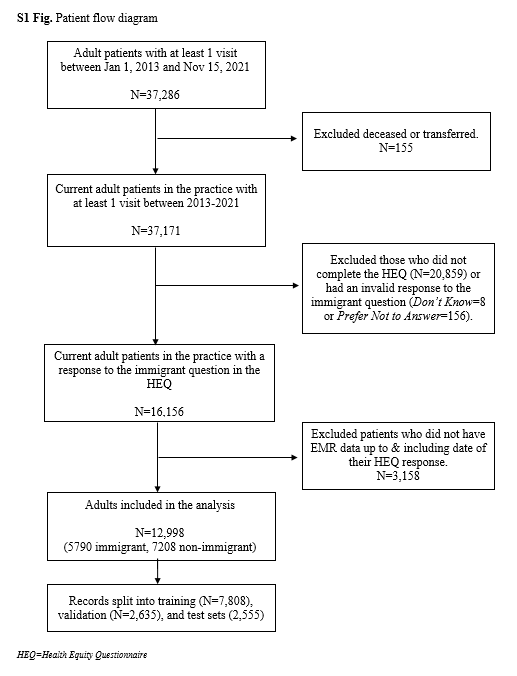

Supplement: S1 Fig — (TIFF) [file pdig.0001336.s001.tiff]

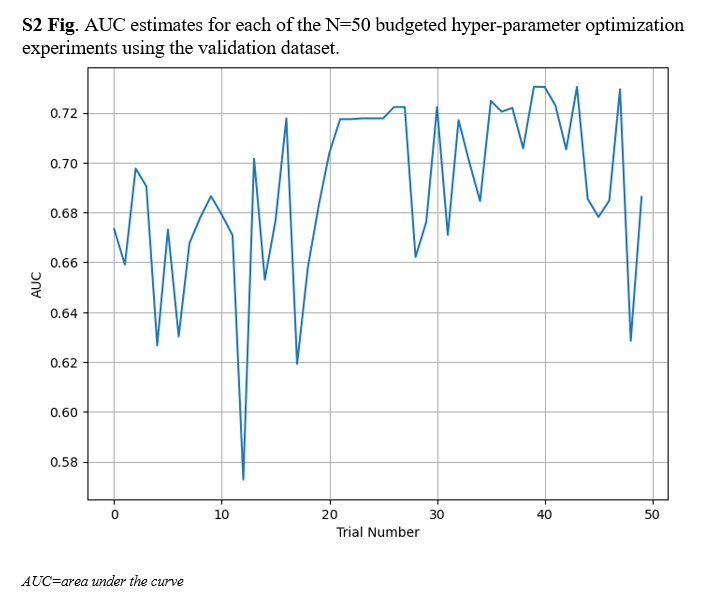

Supplement: S2 Fig — (TIFF) [file pdig.0001336.s002.tiff]
