# Supplementary material for: Comparing regular expression and machine learning approaches to predict immigrant status from primary care electronic medical record data in Ontario, Canada
Source: PLOS Digit Health. 2026 Apr 17;5(4):e0001336. doi: 10.1371/journal.pdig.0001336 (PMC13089691; doi:10.1371/journal.pdig.0001336)
Supplement: S1 Table — (DOCX) [file pdig.0001336.s003.docx]

**S1 Table:** Hyper-parameters associated with the extreme gradient boosting (XGBoost) model, and the generating distributions used to sample hyper-parameter values for each hyper-parameter optimization experiment.

| **Model** | **Hyper-parameter** | **Abbreviation** | **Default Value** | **Generating Distribution** |
| --- | --- | --- | --- | --- |
| Extreme Gradient Boosting Model | Number of Boosting Rounds | “trees” | None | Categorical (100, 250, 500, 1000, 2500, 5000) |
|  | Learning Rate | “lr” | 0.3 | Categorical (1.0, 0.1, 0.01, 0.001, 0.0001, 0.00001) |
|  | Maximum Tree Depth | “depth” | 6 | Categorical (1, 2 ,3, 5, 7, 10, 15) |
|  | Minimum Leaf Weight | “cw” | 1 | Categorical (1) |
|  | Gamma Regularization | “gamma” | None | Categorical (0, 0.1, 1.0) |
|  | Alpha Regularization | “alpha” | 0 | Categorical (0, 0.1, 1.0) |
|  | Lambda Regularization | “lambda” | 1 | Categorical (0, 0.1, 1.0) |
|  | Row Sample Fraction | “rowsample” | 1 | Categorical (0.1, 0.25, 0.50, 1.0) |
|  | Column Sample Fraction | “colsample” | 1 | Categorical (0.1, 0.25, 0.50, 1.0) |
