# Supplementary material for: Comparing regular expression and machine learning approaches to predict immigrant status from primary care electronic medical record data in Ontario, Canada
Source: PLOS Digit Health. 2026 Apr 17;5(4):e0001336. doi: 10.1371/journal.pdig.0001336 (PMC13089691; doi:10.1371/journal.pdig.0001336)
Supplement: S2 Table — (DOCX) [file pdig.0001336.s004.docx]

**S2 Table**: Validation dataset AUC metric, hyper-parameter optimization time, and optimally identified hyper-parameter configurations for the simulated annealing experiments, applied to the binary XGBoost classifier.

| Experiment | Validation AUC | Hyper-Parameter Optimization Time  (Hours) | Optimal Hyper-Parameter Configuration |
| --- | --- | --- | --- |
| Simulated Annealing | 0.731 | 4.73 | trees=2500; lr=0.01; depth=5; cw=1; gamma=0.0; lambda=0.1; alpha=0.1; colsample=0.10; subsample=0.5 |

*AUC=area under the curve*
