# Supplementary material for: Comparing regular expression and machine learning approaches to predict immigrant status from primary care electronic medical record data in Ontario, Canada
Source: PLOS Digit Health. 2026 Apr 17;5(4):e0001336. doi: 10.1371/journal.pdig.0001336 (PMC13089691; doi:10.1371/journal.pdig.0001336)
Supplement: S3 Table — (DOCX) [file pdig.0001336.s005.docx]

**S3 Table:** Discrimination statistics for the XGBoost model stratified by age group and sex.

| **Metric** | **All Patients** | **Males** | **Females** | **Aged 18-39** | **Aged 40-65** | **Aged >65** |
| --- | --- | --- | --- | --- | --- | --- |
| Sensitivity (95% CI) | 53.1  (50.2, 56.0) | 51.5  (46.9, 56.1) | 54.1  (50.4, 57.8) | 52.4  (48.0, 56.8) | 54.8  (50.6, 59.1) | 49.3  (41.3, 57.3) |
| Specificity (95% CI) | 83.2  (81.3, 85.2) | 83.5  (80.5, 86.5) | 83.0  (80.4, 85.6) | 87.0  (84.5, 89.6) | 83.0  (80.0, 86.1) | 67.3  (59.8, 74.8) |
| PPV (95% CI) | 72.6  (69.6, 75.6) | 70.6  (65.7, 75.5) | 73.9  (70.2, 77.7) | 75.2  (70.7, 79.8) | 74.3  (69.9, 78.7) | 60.2  (51.5, 68.8) |
| NPV (95% CI) | 68.0  (65.7, 70.2) | 69.2  (65.8, 72.6) | 67.0  (64.1, 70.0) | 70.9  (67.8, 74.0) | 67.3  (63.9, 70.8) | 57.1  (49.8, 64.4) |
| AUC (95% CI) | 74.6  (72.7, 76.5) | 72.4  (69.2, 75.5) | 76.0  (73.6, 78.4) | 76.5  (73.7, 79.3) | 75.1  (72.2, 78.0) | 62.8  (56.6, 69.1) |
| ICI | 0.039 | 0.057 | 0.043 | 0.055 | 0.050 | 0.063 |
| E50 | 0.031 | 0.048 | 0.022 | 0.048 | 0.042 | 0.067 |
| E90 | 0.115 | 0.106 | 0.119 | 0.139 | 0.133 | 0.106 |
| Emax | 0.136 | 0.155 | 0.133 | 0.160 | 0.151 | 0.115 |

*AUC=area under the curve, CI=confidence interval, ICI=integrated calibration index, NPV=negative predictive value, PPV=positive predictive value*
