# Supplementary material for: Comparing regular expression and machine learning approaches to predict immigrant status from primary care electronic medical record data in Ontario, Canada
Source: PLOS Digit Health. 2026 Apr 17;5(4):e0001336. doi: 10.1371/journal.pdig.0001336 (PMC13089691; doi:10.1371/journal.pdig.0001336)
Supplement: S4 Table — (DOCX) [file pdig.0001336.s006.docx]

**S4 Table:** Discrimination statistics for the REGEX/pattern-matching classifier stratified by age group and sex.

| **Metric** | **All Patients** | **Males** | **Females** | **Aged 18-39** | **Aged 40-65** | **Aged >65** |
| --- | --- | --- | --- | --- | --- | --- |
| Sensitivity (95% CI) | 5.2  (4.0, 6.5) | 4.4  (2.5, 6.3) | 5.8  (4.1, 7.5) | 8.2  (5.8, 10.6) | 3.1  (1.6, 4.6) | 2.7  (0.8, 5.2) |
| Specificity (95% CI) | 99.9  (99.7, 100) | 99.8  (99.5, 100) | 99.9  (99.6, 100) | 99.8  (99.6, 100) | 99.8  (99.5, 100) | 100  (100, 100) |
| PPV (95% CI) | 96.8  (92.5, 100) | 95.2  (86.1, 93.0) | 97.6  (93.0, 100) | 97.6  (93.0, 100) | 94.1  (82.9, 100) | 100  (100, 100) |
| NPV (95% CI) | 55.7  (53.8, 57.7) | 57.7  (54.7, 60.7) | 54.4  (51.8, 57.0) | 59.2  (56.3, 62.0) | 53.6  (50.6, 56.6) | 50.7  (45.0, 56.4) |

*CI=confidence interval; REGEX=regular expression*
