# Supplementary material for: Comparing regular expression and machine learning approaches to predict immigrant status from primary care electronic medical record data in Ontario, Canada
Source: PLOS Digit Health. 2026 Apr 17;5(4):e0001336. doi: 10.1371/journal.pdig.0001336 (PMC13089691; doi:10.1371/journal.pdig.0001336)
Supplement: Attachment — Submitted filename: Response to Reviewers_2025_03-11.pdf [file pdig.0001336.s007.pdf]

Response to Reviewers
March 11, 2025

Comparing regular expression and machine learning approaches to predict immigrant status from primary care electronic medical record data in Ontario, Canada
(Manuscript PDIG-D-24-00471)

Dear Editors,

We thank the reviewers for their time and helpful comments. We have provided our response to their questions and concerns below in blue text.

Reviewer #1

1. The study could explore some data balancing techniques (SMOTE/class weighting) and label validation. Adding more discussions for the FP/FN cases could help to identify patterns in ambiguity, missing context/data, or label noise.

   We agree with the reviewer that SMOTE, over/under-sampling, class weighting, etc. are commonly employed methods for dealing with class imbalance issues and we added this to our Limitations section on page 16. However, recent empirical studies, including van den Goorbergh et al. (2022), have brought into question the impacts of these methods on classification performance (e.g., discrimination can improve but calibration is often degraded). Considering this and given that our outcome (immigrant status) was not significantly imbalanced, we felt that there was little added value to implementing these techniques for this study.

   Van den Goorbergh R, van Smeden M, Timmerman D, Van Calster B. The harms of class imbalance corrections for risk prediction models: illustration and simulation using logistic regression. JAMIA 2022; 29(9):1525-1534. https://doi.org/10.1093/jamia/ocac093

   In terms of adding more discussion about the FP/FN cases, we agree this is a good suggestion and have included more details on page 14. Upon brief review, the false negatives are likely arising from people with the outcome (i.e., immigrant) who are not being identified by the models because information related to this (or relevant predictors) is *not* being documented in the EMR. Conversely, false positives seem to be arising due to 1) measurement error in capturing immigrant status, or 2) semantic ambiguity around terms that are predictive of immigrant status or when the person was born in Canada.

Other metadata, such as time since immigration, country, etc., definitely could have a big impact on the model's performance;

We wholeheartedly agree that metadata related to time since immigration, country, etc. would be extremely valuable. The reference standard (HEQ) asked about whether one was born in Canada (Y/N) and if no, what year they arrived in Canada. However, we decided to exclude this in lieu of a simpler binary classifier, especially since this additional information (year of immigration, country, etc.) is very scarce and not consistently documented within the EMR.

I would suggest including accuracy and F1 score to have a more holistic evaluation.

We agree and have added both balanced accuracy and F1 scores to Table 2 (page 13). We had mainly relied on AUC for inferring discrimination performance, as it is robust, easy to interpret and comes with asymptotic confidence interval estimators. However, it enhances overall interpretability to include both balanced accuracy and F1 scores for a variety of readers.

2. Please correct "e., time since immigration" in Line 366, to "e.g. time since immigration"
   Thank you for catching this error, we have fixed it.

Reviewer #2

1. Selection bias in training data constitutes major limitation of this study. Researchers included patients who completed Health Equity Questionnaire (HEQ), representing portion of total patient population (16,500 out of 37,171 patients). Selective sampling raises concerns about representation, generalizability. Study authors acknowledge individuals who completed surveys might differ from those who didn't. Study population was younger with higher proportion of females compared to clinic population, indicating sampling bias that could affect model's real-world application.

   Yes, we agree that selection bias is a key limitation, largely deriving from respondents/non-respondents to the HEQ, as well as restrictions on dates of EMR data (i.e., to be included in the analytic cohort, there needed to be a HEQ response and EMR data prior). Most patients completed the HEQ between 2013 and 2015 (see table below), when it was first rolled out in the practice; this may have contributed to selection bias. Further, because the HEQ was not mandatory, it may have also impacted who was able to or comfortable with responding and this is a general limitation of surveys. This was the impetus for this study, as the St. Michael's Hospital Family Health Team wanted to explore statistical ways to determine immigrant status for their patients who did not complete the HEQ. We added to this in the Interpretation on pages 14 and 17.

Table: Count and proportion of completed HEQ surveys between 2013 and 2021 (study time period).

Year of HEQ completion	Patients completing HEQ, n (%)
2013	220 (1.3)
2014	5046 (29.4)
2015	4025 (23.4)
2016	1517 (8.8)
2017	1643 (9.6)
2018	2038 (11.9)
2019	1899 (11.1)
2020	668 (3.9)
2021	128 (0.7)

2. Class imbalance handling presents methodological concern. Distribution between classes (44.5% born outside Canada vs 55.5% born Canada) isn't skewed, but researchers don't discuss techniques to address imbalance. Class weights or synthetic minority oversampling technique (SMOTE) could have improved model training. Lack of considerations may have influenced model's performance metrics, sensitivity, specificity, and could explain disparities in prediction accuracy between classes.

   As described above in a previous comment, we do agree that SMOTE, over/under-sampling, class weighting, etc. are commonly employed methods for dealing with class imbalance issues and we added this to our Limitations section on page 17/18. However, recent empirical studies, including van den Goorbergh et al. (2022), have brought into question the impacts of these methods on classification performance (e.g., discrimination can improve but calibration is often degraded). Considering this and given that our outcome (immigrant status) was not terribly imbalanced, we felt that there was little added value to implementing these techniques for this study.

3. Cross-validation methodology shows limitations. Researchers used train-validation-test split (60:20:20) without implementing validation techniques like k-fold cross-validation or repeated sampling. This approach questions stability, reliability of reported performance metrics. Without multiple validation iterations, determining whether results remain consistent across data splits becomes difficult, raising possibility of random split artifacts affecting analysis.

   We agree that there were several different designs that could have been used to evaluate generalization performance, including k-fold cross-validation, other cross-validation or resampling variants, etc. However, we chose to follow a process outlined by Hastie et al. (Elements of Statistical Learning, pg 222;

https://www.sas.upenn.edu/~fdiebold/NoHesitations/BookAdvanced.pdf), where they recommended the best approach for data-rich situations is to randomly divide the dataset into three sets (training, validation, test); "the training set is used to fit the models; the validation set is used to estimate prediction error for model selection; the test set is used for assessment of the generalization error of the final chosen model. Ideally, the test set should be kept in a "vault," and be brought out only at the end of the data analysis. Suppose instead that we use the test-set repeatedly, choosing the model with smallest test-set error. Then the test set error of the final chosen model will underestimate the true test error, sometimes substantially. The training set is used to fit the model, the validation set used to estimate prediction error for model selection, and the test set used for assessment of the generalization error of the final chosen model."

We hypothesize that estimates of model performance would be similar if estimating using a train/validate/test framework (as implemented in our study) vs. k-fold cross-validation. If the dataset was small, then we agree the cross-validation and/or resampling methods may be preferred. However, one consideration with k-fold CV estimates relates to 'averaging metrics over folds' vs. 'concatenating prediction over folds' While the former approach is more commonly employed, in this scenario metric uncertainties seem to be a function of k (number of CV folds) compared to N (sample size of validation/testing samples). This would seem somewhat less than ideal. It was our preference to use concatenated estimators, which rely on binomial sampling models, compared to averaging continuous metrics. The train/validate/test framework avoids these issues, although we acknowledge it does so at some costs (e.g., not using all outcomes to build the model, randomness across splits, etc.).

4. Evaluation metrics, including sensitivity, specificity, PPV, NPV, need expansion. Missing confidence intervals for metrics hinders assessment of estimate precision. Researchers could have included F1 score or balanced accuracy, which suit class imbalance situations. Lack of ROC curves for regular expression approach limits performance comparison of methodologies across decision thresholds.

   We double checked Table 2 and Supplementary Tables 4 and 6 to ensure the metrics were also accompanied by 95% confidence intervals. We have also generated both F1 scores and balanced accuracy metrics and included these in Table 2 (page 13). We did not include ROC curves for the regular expression approach, as this ROC curves generally only apply to probabilistic models (the REGEX approach was a classifier, not probabilistic). For this, we obtained a single estimate of sensitivity and specificity and thus, the ROC curve would be a single point in ROC space.

5. Feature engineering approach shows basic implementation, limiting model's pattern recognition capabilities. Researchers used bag-of-words approach for text features,

missing opportunities for word embeddings implementation. EMR data temporal aspects, structured relationships between medical codes remained unused. Discussion omits feature selection methods, multicollinearity handling between features, raising questions about feature engineering process robustness. Enhanced feature engineering might have improved model performance.

We added a brief summary to the Limitations section (page 16) to note that we did not explore more of these advanced AI approaches in our current study. We were constrained by available resources at the time but we are planning future work that will investigate whether more flexible models, or models with deep capacity/architectures, might be helpful in improving model performance.
